# Identification of PBX1 Target Genes in Cancer Cells by Global Mapping of PBX1 Binding Sites

**DOI:** 10.1371/journal.pone.0036054

**Published:** 2012-05-02

**Authors:** Michelle M. Thiaville, Alexander Stoeck, Li Chen, Ren-Chin Wu, Luca Magnani, Jessica Oidtman, Ie-Ming Shih, Mathieu Lupien, Tian-Li Wang

**Affiliations:** 1 Departments of Pathology and Oncology, Johns Hopkins Medical Institutions, Baltimore, Maryland, United States of America; 2 Department of Gynecology and Obstetrics, Johns Hopkins Medical Institutions, Baltimore, Maryland, United States of America; 3 Department of Electrical and Computer Engineering, Virginia Polytechnic Institute and State University, Arlington, Virginia, United States of America; 4 Institute of Quantitative Biomedical Sciences, Norris Cotton Cancer Center, Dartmouth Medical School, Lebanon, New Hampshire, United States of America; Northwestern University, United States of America

## Abstract

PBX1 is a TALE homeodomain transcription factor involved in organogenesis and tumorigenesis. Although it has been shown that ovarian, breast, and melanoma cancer cells depend on PBX1 for cell growth and survival, the molecular mechanism of how PBX1 promotes tumorigenesis remains unclear. Here, we applied an integrated approach by overlapping PBX1 ChIP-chip targets with the PBX1-regulated transcriptome in ovarian cancer cells to identify genes whose transcription was directly regulated by PBX1. We further determined if PBX1 target genes identified in ovarian cancer cells were co-overexpressed with PBX1 in carcinoma tissues. By analyzing TCGA gene expression microarray datasets from ovarian serous carcinomas, we found co-upregulation of PBX1 and a significant number of its direct target genes. Among the PBX1 target genes, a homeodomain protein MEOX1 whose DNA binding motif was enriched in PBX1-immunoprecipicated DNA sequences was selected for functional analysis. We demonstrated that MEOX1 protein interacts with PBX1 protein and inhibition of MEOX1 yields a similar growth inhibitory phenotype as PBX1 suppression. Furthermore, ectopically expressed MEOX1 functionally rescued the PBX1-withdrawn effect, suggesting MEOX1 mediates the cellular growth signal of PBX1. These results demonstrate that MEOX1 is a critical target gene and cofactor of PBX1 in ovarian cancers.

## Introduction

*Pre-B-cell leukemia homeobox-1* (*PBX1*) belongs to the TALE (three-amino-acid loop extension) family of atypical homeodomain proteins, whose members are characterized by a three-residue insertion in the first helix of the homeodomain [1]. PBX1 protein interacts with other homeodomain-containing nuclear proteins such as HOX and MEIS to form heterodimeric transcription complexes. Biochemical studies have demonstrated that PBX1 and HOX interact through contacts between the PBX1 homeodomain and a conserved hexapeptide sequence in the HOX protein [2]. X-ray crystallographic studies have further demonstrated that PBX1-HOXB1 dimerization is mediated by binding of the HOX hexapeptide to a pocket in PBX1 located between the three-residue insertion and the third helix of the PBX1 homeodomain [3]. The heterodimerization of PBX1 and HOX cooperatively regulates affinity and specificity of their binding to target DNA sequences [4], [5].

Given its critical role in transcriptional regulation, it comes as no surprise that PBX1 is involved in a variety of biological processes from cell fate determination during organogenesis to the development of human cancers [6], [7], [8]. Expression of PBX1 is temporally and spatially regulated to control organ development of spleen, pancreas, kidney, adrenal gland, and skeleton [9], [10], [11], [12], and is implicated in the development of Müllerian duct that later develops into the female genital tract [13].

Emerging results have indicated a role of PBX1 in human cancer. The PBX1-HOX heterodimer complex was found to contribute to oncogenic activity in breast cancer and melanoma. A dominant negative PBX1 protein or HOX hexapeptide motif that disrupted the interaction between PBX1 and HOX was found to significantly reduce the proliferation of these cancer cells [14], [15], [16], [17]. Moreover, PBX1 acts as a pioneer factor in breast cancer, remodeling the chromatin to favor the recruitment of estrogen receptor alpha (ERa) [18]. PBX1 has also been demonstrated to be a downstream effector of the Notch signaling pathway, which is frequently activated in ovarian, cervical, and certain types of breast carcinomas [8], [19], [20]. In light of the potential role of PBX1 in various cancer types, identifying the PBX1 transcription target genes in cancer cells is critical to elucidating its oncogenic mechanisms. In this study, we performed an integrated analysis to identify genes whose promoters were occupied by PBX1 protein and whose expression levels were regulated by PBX1. We have focused on one PBX1 direct target gene, *MEOX1*, and demonstrated its involvement in PBX1-mediated tumor cell growth as well as its direct interaction with PBX1.

## Results

### Global mapping of PBX1 binding sites in ovarian cancer cells

**Figure 1:**
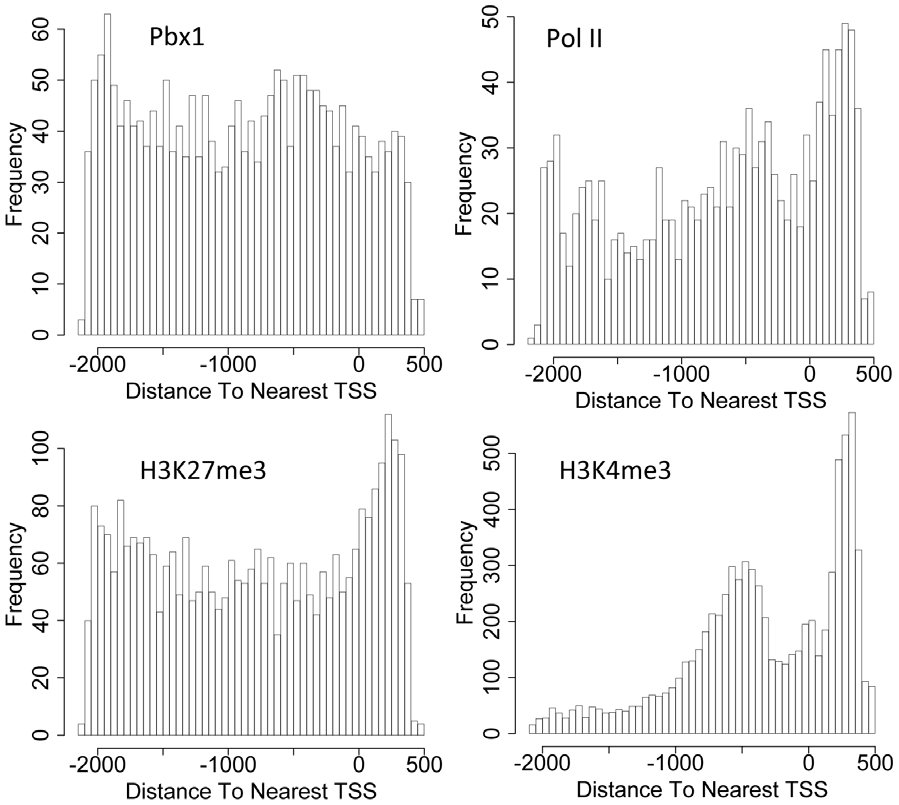
Distribution of PBX1, RNA polymerase II, H3K4me3, and H3K27me3 binding events at gene promoters. Histograms show the distribution of the peak binding frequency with respect to their position on promoters (x axis). Binding frequency as shown in the y axis was determined by the number of peak binding events at specific promoter locations. TSS: transcription start site.

Since PBX1 is a nuclear transcription factor that binds to specific promoters, we sought to identify PBX1 binding sites in the human cancer genome using ChIP-chip analysis. An ovarian cancer cell line, OVCAR3, was selected because this cell line was previously reported to overexpress PBX1, and was dependent on PBX1 expression for cellular survival [8]. PBX1-bound DNA fragments enriched by ChIP were hybridized to a human promoter array containing 18,028 gene promoters, representing a total of 24,659 transcripts. IgG was used as a negative control in a parallel immunoprecipitation. Any positive peaks overlapping those from the IgG control were excluded from the PBX1 peak list. Using a false discovery ratio (FDR)≤0.2, we identified 2,299 unique promoters that showed significant PBX1 binding signals (Table S1). The ChIP-chip data were then used to calculate the PBX1 peak binding distribution across the entire promoter regions, and we found that PBX1 binding sites occurred throughout the promoter regions (Fig. 1). We also performed ChIP-chip to determine the distribution of RNA polymerase II, H3K4me3, and H3K27me3 in promoter regions (Fig. 1), and tested their promoter co-occurrence with PBX1 targets. The results indicated that PBX1 promoter binding co-occurred with all three tested markers, with PBX1-RNA polymerase II being the most significant co-occurrence pair (Table S2). There was no significant co-occurrence between H3K4me3 and H3K27me3, indicating that these two histone marks bound to the promoters of a distinct set of genes.

### Potential cofactors collaborating with PBX1

**Figure 2:**
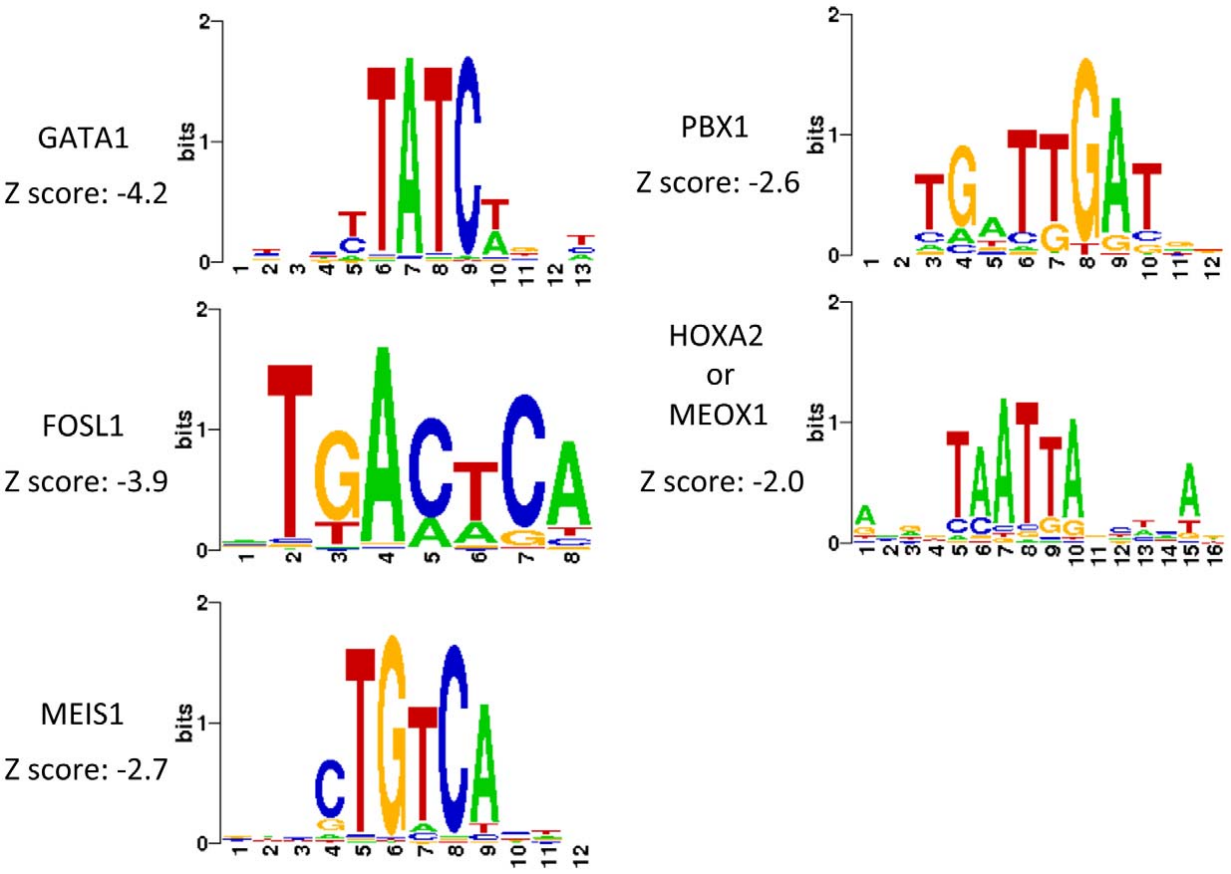
Transcription factor motifs enriched in ChIP-chip target sequences. Selected examples of transcription factor binding motifs that are statistically enriched in the PBX1 immunoprecipitated DNA sequences.

By analyzing DNA sequences enriched by PBX1 ChIP, cis-regulatory cofactors of PBX1 could be identified by scanning DNA binding motifs of the previously characterized transcription factors. The web-based application Cistrome was employed for this analysis (http://cistrome.dfci.harvard.edu/ap/). The canonical PBX1 binding motif was found to be among the significantly enriched sequence motifs (Fig. 2 & Table S3). A plot of the distances between PBX1 binding and its predicted motif revealed a normal (bell-shaped) distribution (Fig. S1). In addition to the PBX1 motif, GATA1, FOSL1, and JUNB transcription factor motifs were significantly enriched, suggesting their potential involvement with PBX1 in transcription regulation. Furthermore, the motif of MEIS1 which is a well-known cofactor of PBX1 and the “TAATTA” motif for both MEOX1 and HOX were also enriched in the PBX1 ChIPed sequences.

### PBX1 directly-regulated transcriptome

Transcription factors can function directly as well as by long-range or indirect mechanisms; thus, to identify genes whose transcription was directly regulated by PBX1, we overlapped the PBX1-regulated transcriptome with the PBX1 ChIP-chip target genes. The PBX1 transcriptome was identified by comparing expression microarray data between PBX1 siRNA-treated and control siRNA-treated OVCAR3 cells. The knockdown efficiency of PBX1 siRNA was validated by Western blot (Fig. S2). We identified 2,094 unique genes whose expression levels were significantly regulated by PBX1 (FDR<0.01). The canonical signaling pathways enriched in the PBX1 transcriptome include Wnt signaling, insulin receptor signaling, and caveolar-mediated endocytosis signaling (Table S4).

**Figure 3:**
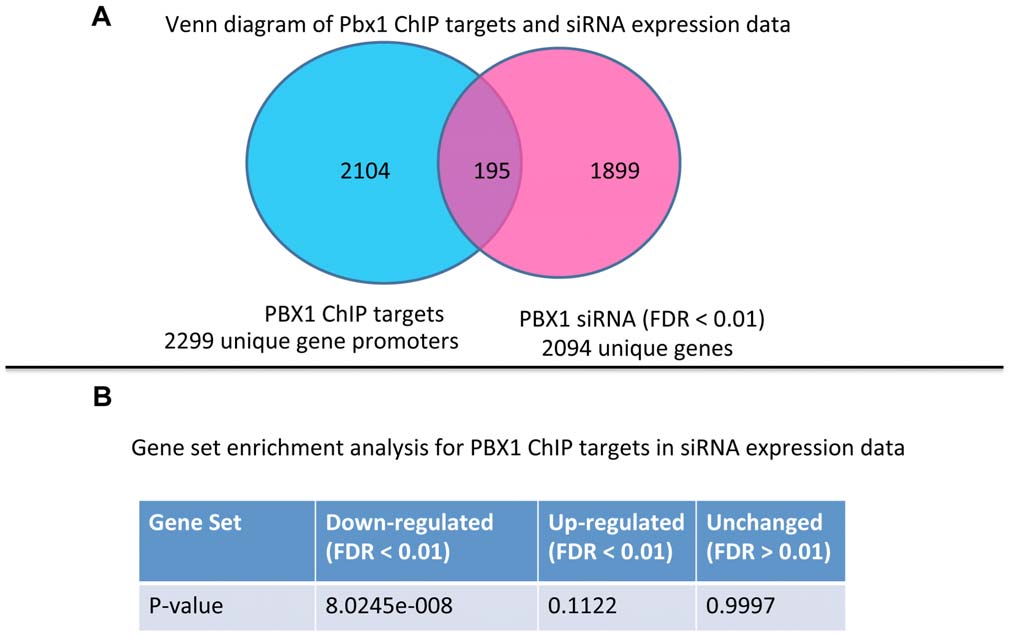
PBX1 binds to a specific set of genes and controls their transcription in ovarian cancer. **A:** Venn diagram of PBX1 ChIP-chip target genes and PBX1 transcriptome reveal a set of overlapping genes that are direct target genes of PBX1. The full list of the overlapping genes is shown in Table S5. Experiments were performed in OVCAR3 cells. **B:** Gene set enrichment analysis was performed to determine if PBX1 ChIP targets are enriched in the PBX1 transcriptome. Genes down-regulated by PBX1 siRNA are significantly enriched in the PBX1 ChIP target set.

Overlapping PBX1 ChIP-chip target genes and the PBX1 transcriptome identified 195 genes that were potential PBX1 direct target genes (Fig. 3A and Table S5). We further analyzed the distribution of PBX1 ChIP target genes with respect to the alterations in the transcriptome in OVCAR3 cells treated with PBX1 siRNA. We were able to show a significant enrichment of PBX1 ChIP targets only among genes down-regulated after PBX1 siRNA treatment (Fig. 3B). In contrast, PBX1 ChIP targets were not enriched in up-regulated genes or in genes whose expression was unchanged (FDR>0.01). To determine potential transcription co-regulation for the PBX1 target genes, the 195 identified genes were overlaid with the ChIP datasets containing modified histone marks (see above). The results demonstrated that the majority (∼70%) of PBX1 target gene promoters also contained H3K4me3 but only ∼2% of PBX1 target gene promoters contained H3K27me3 (Fig. S3 and Column Q in Table S5). Statistical tests demonstrated that the 195 presumptive PBX1 target genes significantly co-occurred with H3K4me3 and RNA polymerase II, but not with H3K7me3 (Table S2). Taken together, these results indicated that PBX1 was required for active expression of the target genes and the promoters of its target genes were often occupied with H3K4me3, a histone mark associated with actively transcribed genes.

**Figure 4:**
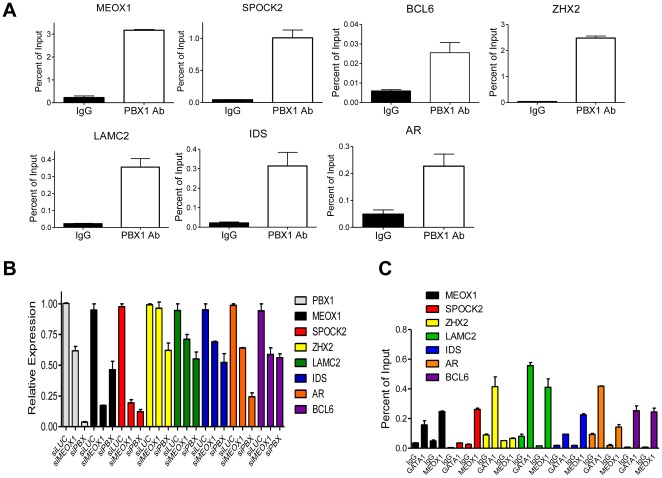
Validation of promoters bound by PBX1 and its cofactors. **A.** Seven genes were chosen from the PBX1 ChIP target gene list, and occupancy of each promoter by PBX1 was validated by ChIP-qPCR analysis. ChIP was performed using either IgG or anti-PBX1 antibody, and the immunoprecipitated DNA was subjected to qPCR analysis using primers flanking the peak of the PBX1 bound region. **B.** Transcriptional regulation of these target genes by PBX1 or MEOX1 was assessed using siRNA and RT-qPCR analysis. Expression of all seven target genes are significantly down-regulated by PBX1 siRNA as compared to control siRNA (Student's *t*-test, *p*<0.01). Except for ZHX2, MEOX1 siRNA significantly downregulated expression of the tested genes (Student's *t*-test, *p*<0.01). **C.** ChIP-qPCR validation of GATA1 and MEOX1 binding near the PBX1-bound sequences. ChIP was performed using IgG, anti-GATA1, or anti-MEOX1 antibody and the immunoprecipitated DNA was subjected to qPCR using same primers as in **A**. Except for binding of MEOX1 to the *ZHX2* promoter, binding of GATA1 or MEOX1 to gene promoters is significantly higher than binding of IgG to the same promoters (Student's *t*-test, *p*<0.01).

Seven representative candidates, based on their known functions in cancer biology, were selected from the 195 candidate PBX1 targets for validation using qPCR. The results confirmed specific binding of PBX1 to the promoters analyzed (Fig. 4A), and confirmed transcript down-regulation in response to PBX1 knockdown for each of these analyzed genes (Fig. 4B).

To determine if potential PBX1 cofactors identified from the motif analysis described above co-occur with PBX1 binding in promoter regions, we performed ChIP-qPCR for GATA1 and MEOX1 using PCR primers flanking the peak binding sequences of PBX1. Western blot analysis demonstrated that both transcription factors were expressed in OVCAR3 cells (Fig. S4A). ChIP-qPCR demonstrated that GATA1 bound to all the tested promoters and MEOX1 was present in 6 of 7 tested promoters (Student's *t*- test, *p*<0.01; Fig. 4C).

### Correlation of PBX1 and target gene expression in ovarian cancer tissues

**Figure 5:**
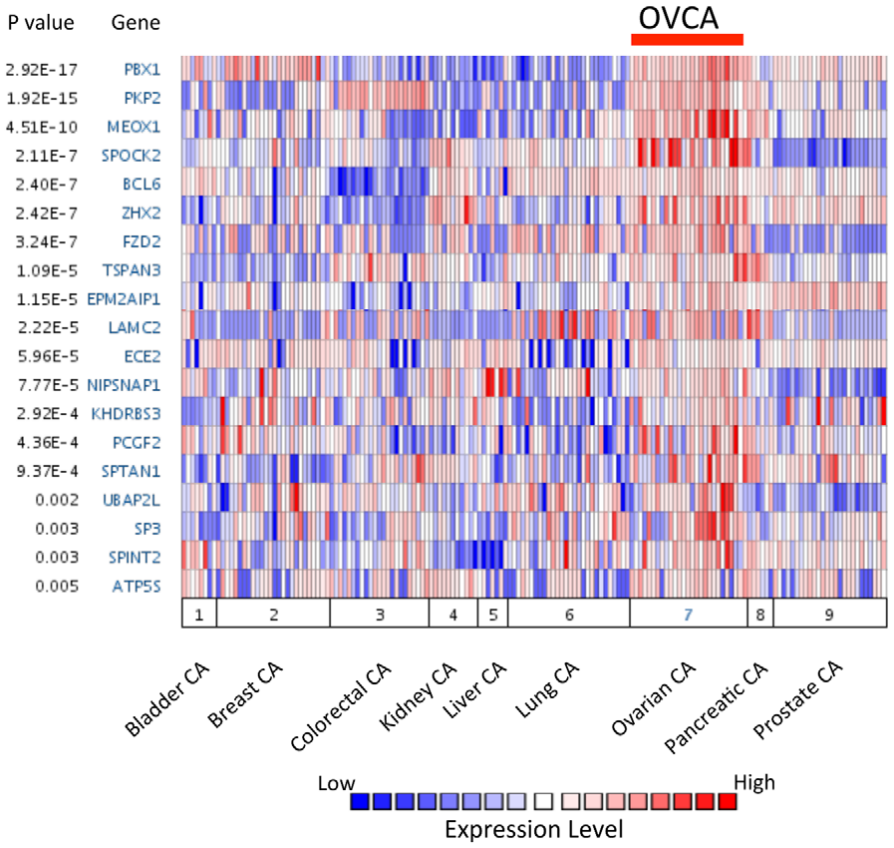
PBX1 and several of its direct target genes are over-expressed in ovarian cancer tissues. Oncomine meta-analysis was performed on a previously published gene expression microarray dataset containing nine different tumor types. Genes were ranked according to their over-expression in ovarian cancer and the top 18 genes were plotted.

To extrapolate the biological significance of the PBX1 target genes identified in the OVCAR3 cell line with respect to human cancer tissues, we performed *in silico* analysis using a publicly accessible expression microarray dataset [21] to determine if PBX1 expression was correlated with its target genes in 9 different types of carcinoma tissues. A total of 86 candidate PBX1 target genes were identified in the microarray dataset [21], and these genes were queried in Oncomine. The results demonstrated that ovarian carcinoma has a high level of PBX1 expression as compared to the other 8 types of cancers (Fig. 5). Furthermore, 28 of the 86 queried genes were significantly up-regulated in the ovarian cancers as compared to other carcinomas (*p*<0.05).

The recently available TCGA ovarian serous cancer dataset permitted us to perform statistical analysis to determine the co-upregulation of PBX1 and its target genes in ovarian carcinomas. Among 195 potential PBX1 target genes, further analysis was performed on a total of 137 for whom gene expression data were present in all three different microarray platforms available from the cBio Cancer Genomics Portal (http://www.cbioportal.org/public-portal/). Among these 137 PBX1 target genes, 53 genes showed a tendency toward co-upregulation with PBX1 (odds ratio >1.5), of which 18 (13.1%) were significant (*p*<0.05). This is higher than would be expected by chance (*p* = 0.013, Chi-square test), because applying the same analysis to the entire set of 11,201 genes available for analysis revealed that only 873 (7.8%) genes showed both an odds ratio greater than 1.5 and *p*<0.05. The odds ratio and significance (*p*-value) of co-upregulation of PBX1 and its target genes are shown in Table S5 columns H and I, respectively.

### MEOX1 directly interacts with PBX1 and rescues the PBX1-withdrawn effect

**Figure 6:**
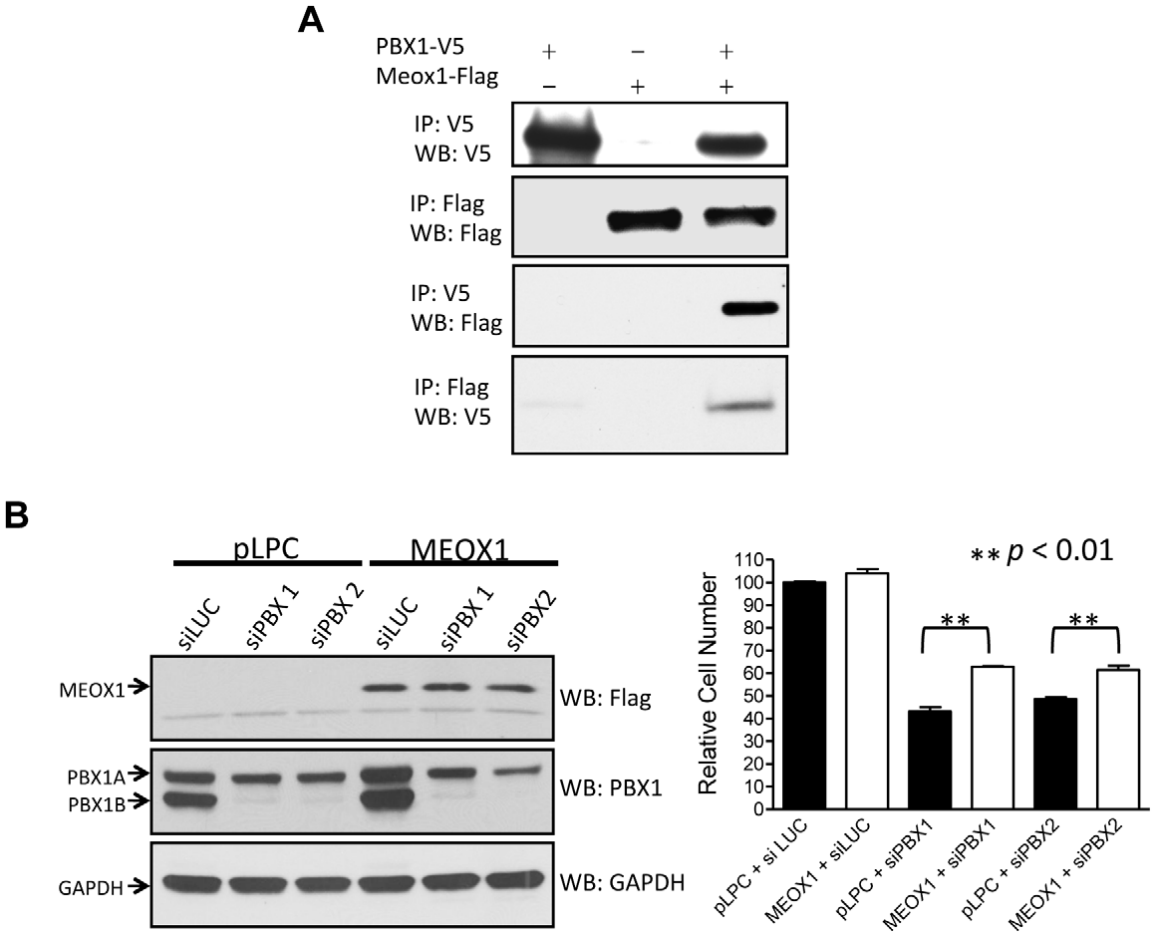
MEOX1 interacts with PBX1 and mediates its growth effect. **A.** HEK293 cells were transfected with PBX1-V5 and/or MEOX1-FLAG expression vectors. Immunoprecipitation and Western blot were performed using epitope tag-specific antibodies. **B.** Left panel: OVCAR3 cells were transfected with MEOX1 expression vector or control vector. Western blot was performed to test MEOX1 expression (top). The same cells were transfected with PBX1 siRNA or control siRNA and Western blot was performed to test the down-regulation of PBX1 protein (middle). Detection of GAPDH protein was used as a loading control (bottom). Right panel: Relative cell numbers were measured in OVCAR3 cells transfected with MEOX1 cDNA, PBX1 siRNA, control plasmid (pLPC), and control siRNA (siLuc). Student's *t*-test was used to determine the significance between the MEOX1 over-expressed group and the control group.

We then selected *MEOX1*, one of the direct targets of PBX1, for further functional studies. Like PBX1, MEOX1 is a homeodomain protein and can potentially interact with PBX1 to form heterodimeric transcription complexes. Thus, we asked if MEOX1 and PBX1 physically interacted in cells. A co-immunoprecipitation experiment was performed in HEK293 cells transfected with PBX1-V5 and MEOX1-FLAG cDNA expression vectors. The results demonstrated that PBX1 pull-down using a V5 antibody significantly enriched MEOX1 protein detected by Western blot with a FLAG antibody (Fig. 6A). Reciprocal co-immunoprecipitation confirmed that MEOX1 co-immunoprecipitated with PBX1 (Fig. 6A).

We also tested a panel of PBX1-regulated genes to determine if MEOX1 was involved in regulating their transcription. Using MEOX1 siRNA, we found that expression of six of the seven PBX1-regulated genes was also dependent on MEOX1. In addition, we found that *PBX1* transcription was dependent on MEOX1, as MEOX1 siRNA decreased the *PBX1* mRNA level. Reciprocally, the *MEOX1* mRNA level was down-regulated by PBX1 knockdown. Previous studies have demonstrated that PBX1 was essential for cell growth in ovarian cancer cells including OVCAR3; here, we performed siRNA knockdown to determine if MEOX1 knockdown produced a phenotype similar to PBX1 knockdown. The results demonstrated that MEOX1 knockdown significantly reduced cell growth in OVCAR3 cells, a phenotype resembling the PBX1-withdrawn effect (Fig. S5).

To test if MEOX1 mediated the cell growth function of PBX1, we expressed MEOX1 constitutively (driven by a CMV promoter) and suppressed PBX1 expression using siRNA. We found that constitutive MEOX1 expression partially protected cells from PBX1 withdrawal-induced growth suppression, as significantly greater cell numbers were observed in MEOX1-transfected cells compared to control plasmid-transfected cells (Fig. 6B). These findings suggest that MEOX1 is an essential mediator of the PBX1-related growth signal, although expression of MEOX1 itself is not sufficient to stimulate cellular proliferation (Fig. S6).

## Discussion

The homeobox gene family, which is critical in transcriptional regulation, encodes nuclear proteins containing highly conserved DNA-binding homeodomains [22]. Homeobox proteins are involved in critical activities during development and tumorigenesis. In this report, we focused on one of the homeobox genes, PBX1, which was found to play a critical role in ovarian cancer, with the intent of identifying and characterizing its transcriptional network in cancer cells. Integrating analysis of gene promoters bound by PBX1 and the PBX1 transcriptome allowed us to identify genes directly regulated by PBX1.

The robustness of our genome-wide ChIP-chip assay is evidenced by the observation that the canonical PBX1 motif is highly enriched in the PBX1 ChIPed sequences. We have demonstrated that 195 PBX1 ChIP targets significantly overlapped with promoter occupancy of RNA polymerase II and H3K4me3, a histone mark associated with active transcription, but not with H3K27me3, a histone mark associated with transcription repression. In addition, we found that ChIP targets were significantly enriched only among genes whose transcription is supported by PBX1 (downregulated by PBX1 siRNA) but not among genes whose transcription is suppressed or not affected by PBX1. These data taken together support the view that PBX1 primarily serves as a transcriptional activator rather than a repressor in ovarian cancer cells.

The current study also revealed potential cis-regulatory cofactors of PBX1, which include GATA1, FOSL1, MEIS1, JUNB, and a “TAATTA” motif for MEOX1 [23] and HOX [24]. The motifs of these transcription cofactors were significantly enriched in PBX1-bound sequences, suggesting that these proteins may work in concert with PBX1 to facilitate transcriptional regulation. Interestingly, several of these potential PBX1 cofactors, including BCL6 and MEOX1, were also identified being directly regulated by PBX1, suggesting an auto-regulatory transcriptional control of these genes. The current study focused on MEOX1 to assess its co-binding and co-regulatory activity with PBX1. Further experiments should be performed to determine if other cofactors serve as co-regulatory factors with PBX1 in cancer cells.

Another exciting finding in this study is the identification of a new role of MEOX1 in cancer pathogenesis. Although MEOX1 is well-established as one of the key transcriptional regulators during embryonic development, our results demonstrate that MEOX1 is also involved in cancer development through participating in the PBX1 transcriptional network. The fact that MEOX1 is co-upregulated with PBX1 in a subset of ovarian carcinomas (Fig. 5) suggests that a PBX1-MEOX1 molecular axis is involved in the transcriptional regulation of these carcinomas. Based on this rationale, we performed a “rescue” assay by ectopically expressing MEOX1 in cancer cells with PBX1 knockdown. Our result showing that MEOX1 expression reversed the growth inhibitory effect of PBX1 knockdown suggests that MEOX1 mediates the growth-supporting function of PBX1 in ovarian cancer cells. Both PBX1 and MEOX1 belong to the homeodomain family, whose members usually interact with other homeodomain proteins to generate heterodimeric protein complexes that are the functional mediators of transcriptional regulation. Indeed, our co-immunoprecipitation experiment demonstrated interaction of PBX1 and MEOX1 proteins, suggesting that MEOX1 can be a critical homeodomain cofactor of PBX1. Thus, PBX1 appears to act not only as an upstream regulator but also as a binding cofactor of MEOX1. This finding is reminiscent of a previous study showing that PBX1 and HOXB1 cooperate in regulating HOXB1 expression activity in mouse tissues [25]. It can be envisioned that this positive auto-regulatory loop operates to ensure sustained transcriptional activity of other PBX1 regulated genes.

The results as reported here represent a first step toward understanding the complex regulatory network of PBX1 in carcinomas. As the role of PBX1 in human solid tumors is emerging, the target genes directly controlled by PBX1 reported herein will serve as footsteps for deciphering how PBX1 contributes to tumor development. This study also raises several important questions yet to be addressed. For example, it would be interesting to test whether the PBX1 regulated genes identified in ovarian carcinoma are shared with the machinery operating during organ development. Since overexpression of PBX1 and MEOX1 is relatively specific to ovarian cancer as compared to other solid tumors (Fig. 5), the biological significance of PBX1 and MEOX1 co-expression and their cooperation in transcriptional regulation are likely to be context- and tissue-dependent. From this perspective, it would be important to determine if MEOX1 modulates the selectivity and target sequence binding affinity of the PBX1 transcription complex in ovarian cancer, and ultimately, it would be interesting to determine how PBX1/MEOX1 complex formation promotes oncogenesis.

## Materials and Methods

### Cell Lines

The cell lines used in this study included an ovarian cancer cell line, OVCAR3, and an embryonic kidney epithelial cell line, HEK293. Both cell lines were purchased from the American Type Culture Collection (ATCC, Manassas, Virginia). The cell lines were maintained in RPMI1640 supplemented with 5% fetal bovine serum and antibiotics. There was no evidence of mycoplasma contamination based on a PCR assay.

### Chromatin Immunoprecipitation (ChIP) Analysis

For each immunoprecipitation, approximately 12×10^6^ OVCAR3 cells were used. Cells were plated in 150 mm dishes and cultured until 80% confluent. Cells were fixed by the addition of formaldehyde (1% final concentration) to culture media and incubation for 10 min. The reaction was quenched with 125 mM glycine for 5 min. Cells were washed in DPBS, nuclei were swollen in nuclei swelling buffer (5 mM PIPES pH 8.0, 85 mM KCl, 0.5% NP 40), and lysed in nuclei lysis buffer (1% SDS, 10 mM EDTA, 50 mM Tris-HCl, pH 8.0). Lysed nuclei were sonicated in a Fisher model 100 sonicator for 5 cycles of 40 sec constant pulse with 2 min ice incubation in between pulses. Sonicated lysates were diluted in ChIP dilution buffer (0.01% SDS, 1.1% Triton X-100, 1.2 mM EDTA, 16.7 mM Tris-HCl, pH 8.0, 167 mM NaCl) and incubated with Protein G Sepharose (Invitrogen) for 1 hr. Beads were removed, and bead cleared lysates were incubated with antibodies against PBX1, GATA1, or MEOX1 (Santa Cruz sc-899, sc-265×, and Epitomics T2204, respectively) overnight with rotation at 4°C. A 1∶1 slurry of BSA-blocked Protein G Sepharose was added to antibody incubated lysates and rotated at 4°C for 2–3 hours. Antibody-protein complexes bound to beads were washed once with Low Salt buffer (0.1% SDS, 1% Triton X-100, 2 mM EDTA, 20 mM Tris-HCl, pH 8.0, 150 mM NaCl), once with LiCl buffer (0.25 M LiCl, 1% NP 40, 1% Sodium Deoxycholate, 1 mM EDTA, 10 mM Tris-HCl, pH 8.0), and twice with TE, pH 8.0. Complexes were eluted from beads by incubating in 1% SDS, 0.1 M NaHCO_3_ (heated to 65°C) for 30 min at 37°C with shaking. Samples were treated with 200 mM NaCl and 1 µg RNase A at 65°C overnight. After 1 hr proteinase K treatment at 37°C, DNA was purified using the Qiagen QIAquick PCR purification kit and eluted in 100 µl TE.

For qPCR analysis, 2.5 µl of sample was used per reaction. PCR was performed using SyBr Green dye and an iCycler (Bio-Rad, Hercules, CA). The threshold cycle numbers (Ct) were obtained using the iCycler Optical system interface software. PCR primers were designed using the Primer 3 program (nucleotide sequences of the primers are shown in Table S6). Data were normalized using a standard curve, which was prepared from sonicated input (total) DNA.

### ChIP-chip Array Analysis

For ChIP-chip array analysis, ChIP was performed as described above except for the DNA purification step. DNA was purified using the Qiagen MinElute PCR purification kit and eluted with 10 µl H2O. The entire immunoprecipitated DNA sample was amplified using a WGA2 kit (Sigma) and purified using the Qiagen QIAquick PCR purification kit with an elution volume of 40 µl in H2O. For input DNA, 200 ng of purified DNA were used for WGA2 PCR. An aliquot of each amplified sample was diluted to 5 ng/µl and used for qPCR. If 4 µg of DNA were not produced from the WGA2 PCR, 10 ng of the WGA2 reaction were reamplified using the WGA3 kit (Sigma), and tested with control primers again. Aliquots of each sample (4 µg) were sent to NimbleGen for labeling and array hybridization. The NimbleGen 385K RefSeq promoter 1-plex array was used for sample hybridization.

For array analysis, the NimbleGen program Signal Map was used to obtain signal peaks for each sample and to map these peaks to their corresponding genomic regions. Using the Bioconductor package GenomicRanges (available at bioconductor.org), we screened the peak regions from target ChIP-chip with FDR≤0.2 for overlap with peaks from non-specific IgG ChIP-chip and discarded those peaks with at least one bp overlap. The remaining peaks from target ChIP-chip were considered positive for protein binding.

Motif search analyses were performed using the seqpos motif discovery tool (Cistrome, http://cistrome.org/ap/root) and the TRANSFAC motif database with the following parameter: width from the peak to be scanned = 600 bp; *p*<0.05 was set as the cutoff for significance.

### Gene Expression Array Analysis

RNA was isolated from OVCAR3 cells that were previously treated with either PBX1 siRNA or control siRNA targeting luciferase gene. RNA labeling, hybridization to the Illumina human HT-12 Expression Array, and normalization analysis were performed by the Lowe Family Genomics Core facility, Johns Hopkins Bayview Medical Center. The fold change of expression level of each gene was calculated as the ratio of PBX1 siRNA treatment to control. As there was only one array at each time point, we used the logarithm of fold change as the test statistic, and conducted significance analysis to calculate the *p*-value, which was defined as the probability of obtaining a test statistic at least as extreme as the one actually observed under the null hypothesis. For null distribution we assumed the test statistic followed a normal distribution where the mean and standard deviation were estimated from the control samples. We also implemented Benjamini and Hochberg's procedure [26] for multiple hypothesis testing, and estimated the false discovery rate (FDR) for significantly expressed genes. Significantly up-regulated and down-regulated genes were finally determined by a predefined FDR cutoff<0.01.

### Statistical test for gene overlapping among array datasets

To evaluate the significance of overlap between datasets (i.e. between ChIP-chips and between 195 target genes and ChIP-chip), we determined the probability of obtaining the same number or more overlapping genes by randomly sampling from the full set of genes. The number of genes in the full set was 18,511 for ChIP-chip and 30,500 for expression array. The *p*-value was calculated by determining the upper tail probabilities of the corresponding hypergeometric distribution using phyper() function in R version 2.14.1.

### Statistical test for co-expression between PBX1 and its target genes

The Z-scores of mRNA expression data from the TCGA ovarian cancer study were retrieved from the Cancer Genomics Data Server (CGDS) hosted by Memorial-Sloan-Kettering Cancer Center (http://www.cbioportal.org/) using the CGDS-R 1.1.19 package (http://cran.r-project.org/web/packages/cgdsr/index.html). The Z-scores were calculated using diploid tumors (diploid for each gene) as the reference population, and only genes represented by all three expression platforms used in the TCGA study were included [27]. A total of 11,202 genes from 316 tumor samples with Z-scores were retrieved for analysis.

Over-expression for each gene was defined by a Z-score>1.65 (representing the top 5% in mRNA expression). The tendency of co-overexpression of PBX1 and its target genes in the same sample was assessed in the 316 tumor sample set, and odds ratios (ORs) were calculated. Following the criteria used for co-overexpression test on cBio Cancer Genomics Portal (http://www.cbioportal.org/), we considered two genes to have a tendency toward co-overexpression if their odds ratio was greater than 1.5. The significance of co-overexpression was further tested using a one-sided Fisher's exact test.

### Gene knockdown and Quantitative Real-time PCR

PBX1-specific small interfering RNAs (siRNAs) (CCCAGGUAUCAAACUGGUUUGGAAA) and (AAGCCUGCCUUGUUUAAUGUG) and control siRNA targeting the luciferase gene (UAAGGCUAUGAAGAGAUA) were purchased from Invitrogen or synthesized by Integrated DNA Technologies (Coralville, IA). MEOX1-specific siRNAs were purchased from Invitrogen (CCAGGAGGAGCACAUCUUCACUGAG and CACUGCCAAUGAGACAGAGAAGAAA). Cells were treated with siRNA at a final concentration of 100 nM using Lipofectamine RNAiMax according to the manufacturer's suggestion (Invitrogen, Carlsbad, CA). Forty-eight hours after transfection, cells were harvested for RNA isolation using a Qiagen RNeasy kit. cDNA was synthesized using 500 ng RNA according to the iScript cDNA Synthesis kit (Biorad). The cDNA was diluted to a final concentration of 200 ng/µl, and 1 µl was used for each qRT-PCR reaction. Relative gene expression was measured by quantitative real-time PCR using SyBr Green dye and an iCycler (Bio-Rad). The iCycler Optical system software was employed to determine the threshold cycle numbers (Ct). PCR primers were designed using the Vector NTI program (nucleotide sequences of the primers are shown in Table S7). Mean Ct of the gene of interest was calculated from duplicate measurements of at least three separate samples, and was normalized with the mean Ct of a control gene, glyceraldehyde-3-phosphate dehydrogenase (GAPDH), for which expression is relatively constant.

### In silico analysis of PBX1 gene expression among different tumor types

In order to assess the correlation of expression levels in PBX1 and its downstream targeted genes in different major types of human cancer, we performed an *in silico* analysis using Oncomine, which provided cancer microarray databases and integrated data-mining platform [28]. Genes identified as PBX1 targets by ChIP-chip and microarrays were queried in Oncomine to identify associated gene expression signatures with the following threshold: OR = 2 and *p*-value<10^−4^. Associated signatures were filtered for studies including different cancer types. A heat map was generated where rank indicates the genes with the highest association with a particular cancer according to *p*-value based on *t*-test. Specifically, we compared differential gene expression (fold changes) between ovarian cancer and other cancer types.

### Co-Immunoprecipitation and Western Blotting

The generation of a PBX1-V5 expression vector was previously described [8]. The MEOX1-FLAG expression vector was created by subcloning the MEOX1 cDNA (Ultimate ORF clone ID: IOH40231, Invitrogen) into the pLPC-N-FLAG destination plasmid using the Gateway LR Clonase II enzyme mix following the manufacturer's protocol (Invitrogen). Expression plasmids were transfected into HEK293 cells using Lipofectamine 2000 (Invitrogen) and 24 h after transfection, cells were harvested for co-immunoprecipitation experiments. For this, cells were lysed in lysis buffer (50 mM Tris, ph 8.0, 150 mM NaCl, 1% NP40 supplemented with protease inhibitor cocktail (Thermo Scientific)). Lysates were then incubated with either anti-V5 agarose (Sigma, St. Louis, MO), or anti-FLAG M2 affinity gel (Sigma, St. Louis, MO). After five washes (3 times with lysis buffer and 2 times with TBS), precipitates were resuspended in Laemmli sample buffer containing 5% β-mercaptoethanol. Samples were separated by SDS-PAGE, and transferred onto a PVDF membrane using a semi-dry transfer apparatus (Bio-Rad). The membrane was blocked with 5% non-fat dry milk in TBST (20 mM Tris-HCl, 0.5 M NaCl, 0.1% Tween 20) and incubated with a primary antibody, followed by washes with TBST. Subsequently the membrane was incubated with horseradish peroxidase-conjugated secondary antibody (Jackson Laboratories, West Grove, PA) and detected with ECL developing solution (Thermo Scientific). Western blot was performed using an anti-FLAG antibody (Sigma, St. Louis, MO) to detect MEOX1 protein or an anti-V5 antibody (Bethyl, Montgomery, TX) to detect PBX1 protein. To assure equal loading of proteins from cellular extracts we probed membranes with a rabbit polyclonal anti-GAPDH antibody (Sigma).

### Cellular Growth Assay

MEOX1-expressing retrovirus was generated by transfecting MEOX1-pLPC-N-FLAG plasmid into the Phoenix amphotropic packaging cell line (gift from Dr. Garry P. Nolan, Stanford University, CA) as previously described [8]. Viral stock with empty vector (pLPC-N-FLAG) was also generated to serve as control. For retroviral transduction, OVCAR3 cells were seeded into 25-cm^2^ tissue culture flasks and cultured for 24 h prior to infection. Cells were incubated with 4 ml of viral stock overnight in the presence of 8 µg/ml polybrene. Viral supernatant was removed and cells were incubated in regular growth media containing 3 µg/ml puromycin to establish stable clones. Cells stably expressing MEOX1 or empty plasmid (pLPC) were transfected with PBX1 specific siRNA or control siRNA using the RNAiMAX transfection reagent (Invitrogen) according to the manufacturer's protocol. Cells were plated in 96-well plates at 5×10^3^ cells/well 24 h after transfection. The cell number was determined 48 h after plating based on the fluorescence intensity of SYBR green I nucleic acid staining (Molecular Probes, Eugene, OR) measured in a fluorescence microplate reader (Fluostar from BMG, Durham, NC). The data were expressed as mean ± SD from triplicate values.

## Supporting Information

Figure S1 **Distribution of distance between PBX1 peak binding and the closest PBX1 motif at each promoter.**
(PDF)

Figure S2 **Western blot analysis of PBX1 siRNA-treated OVCAR3 cells.** Cells were harvested 48 hours after siRNA treatment. Each siRNA sample was loaded in duplicate for analysis.
(PDF)

Figure S3 **Distribution of two histone methylation marks among 195 PBX1 target genes.**
(PDF)

Figure S4 **Western blot analysis demonstrates the expression of GATA1 and MEOX1 in OVCAR3 cells.**
(PDF)

Figure S5 **Knockdown of MEOX1 inhibits cell growth of OVCAR3 cells.** **A.** Western blot demonstrates the knockdown efficiency of two MEOX1 siRNAs. siRNA against luciferase (siLuc) was used as a control. The cell lysate from each siRNA treatment was loaded onto two lanes of an SDS polyacrylamide gel. **B.** Relative cell numbers were determined by the fluorescence intensity measured at different time points compared to that measured on day 0.
(PDF)

Figure S6 **Cellular proliferation assay performed in OVCAR3 cells stably expressing MEOX1.** Relative cell number was determined by the fluorescence intensity measured at different time points compared to the fluorescence intensity measured on day 0.
(PDF)

Table S1 **Gene promoters with significant PBX1 binding identified by ChIP-chip.**
(XLSX)

Table S2 **Statistical test of co-occurrence between ChIP-chip datasets and between 195 targets and ChIP-chip dataset.** Numbers represent p-values.
(XLSX)

Table S3 **Canonical transcription factor motifs enriched in PBX1 ChIPed sequences.**
(XLSX)

Table S4 **Ingenuity analysis of PBX1 transcriptome.**
(XLSX)

Table S5 **Genes identified by intersecting PBX1 ChIP-chip and transcriptome datasets.**
(XLSX)

Table S6 **Primers for ChIP qPCR analysis.**
(XLSX)

Table S7 **PCR primers for mRNA analysis.**
(XLSX)
