# Supplementary figures and images for: Identification of PBX1 Target Genes in Cancer Cells by Global Mapping of PBX1 Binding Sites
Source: PLoS One. 2012 May 2;7(5):e36054. doi: 10.1371/journal.pone.0036054 (PMC3342315; doi:10.1371/journal.pone.0036054)

Fig. S1

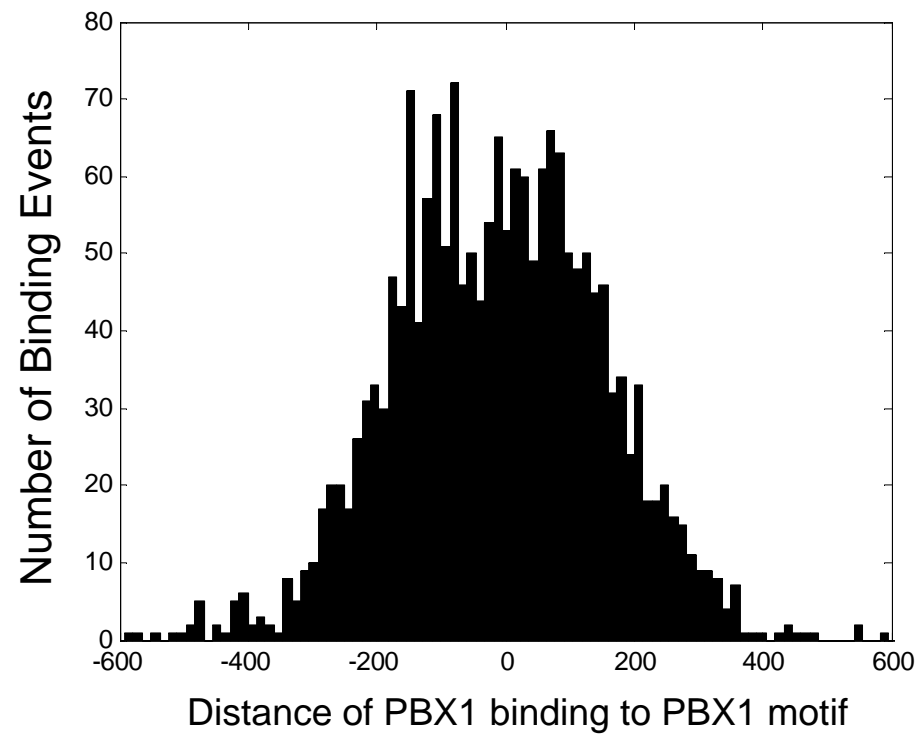

Supplement: Figure S1 — Distribution of distance between PBX1 peak binding and the closest PBX1 motif at each promoter. (PDF) [file pone.0036054.s001.pdf]

Fig. S2

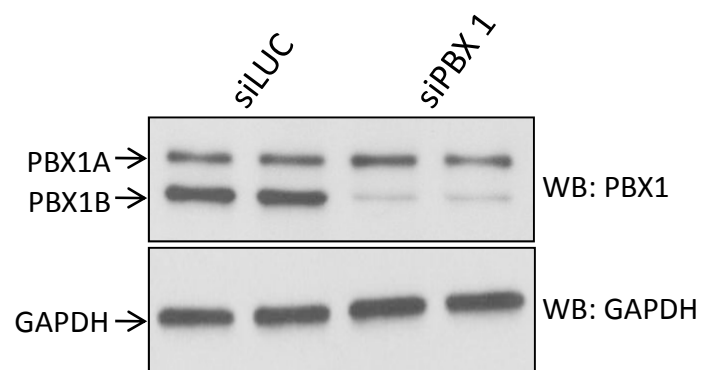

Supplement: Figure S2 — Western blot analysis of PBX1 siRNA-treated OVCAR3 cells. Cells were harvested 48 hours after siRNA treatment. Each siRNA sample was loaded in duplicate for analysis. (PDF) [file pone.0036054.s002.pdf]

Fig. S3

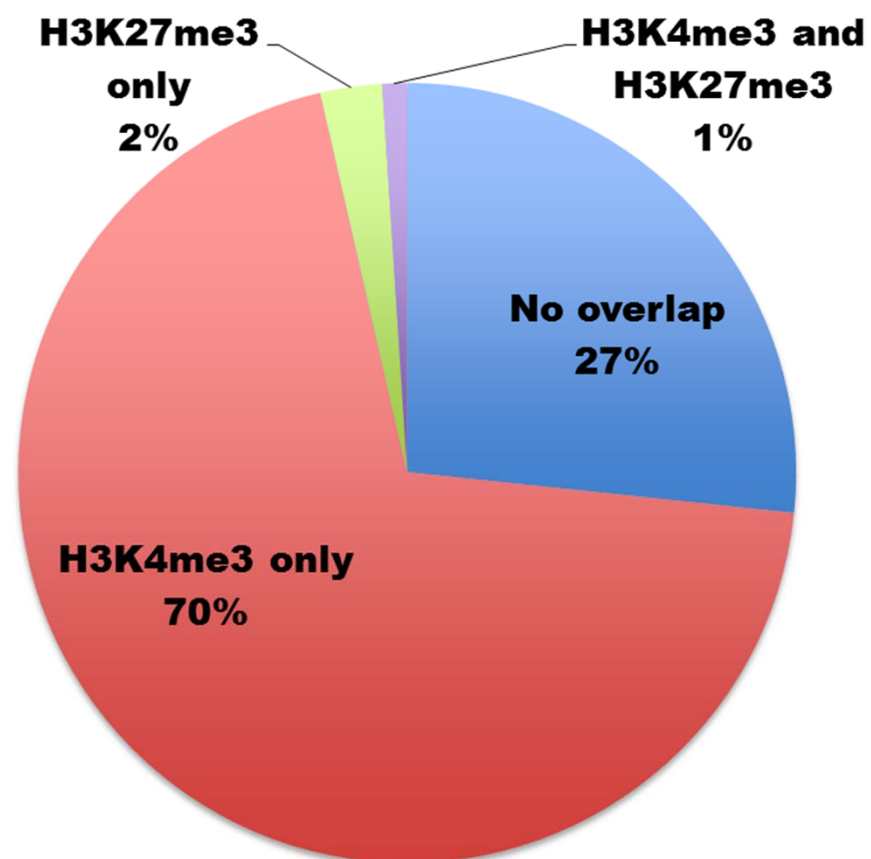

Supplement: Figure S3 — Distribution of two histone methylation marks among 195 PBX1 target genes. (PDF) [file pone.0036054.s003.pdf]

Fig. S4

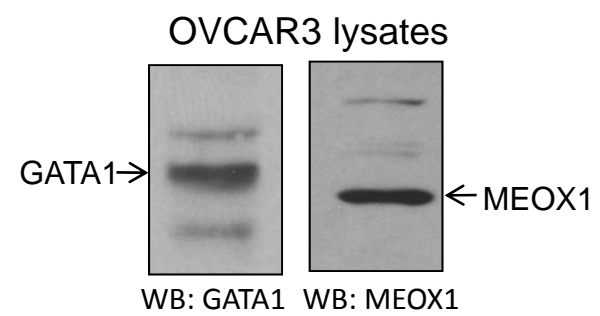

Supplement: Figure S4 — Western blot analysis demonstrates the expression of GATA1 and MEOX1 in OVCAR3 cells. (PDF) [file pone.0036054.s004.pdf]

Fig. S5

**A**

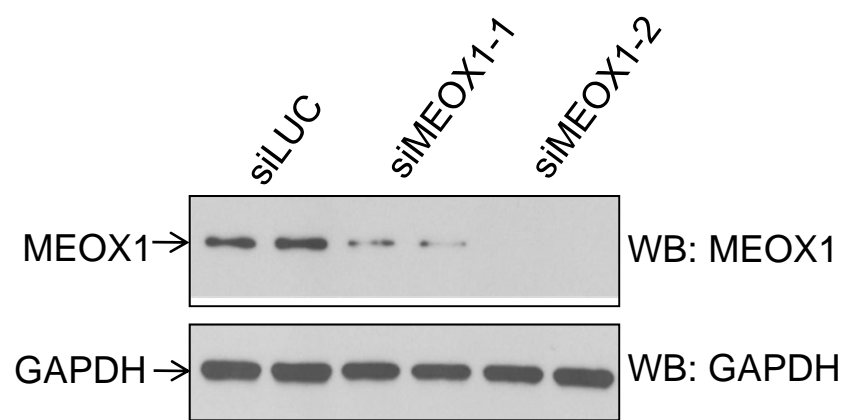

**B**

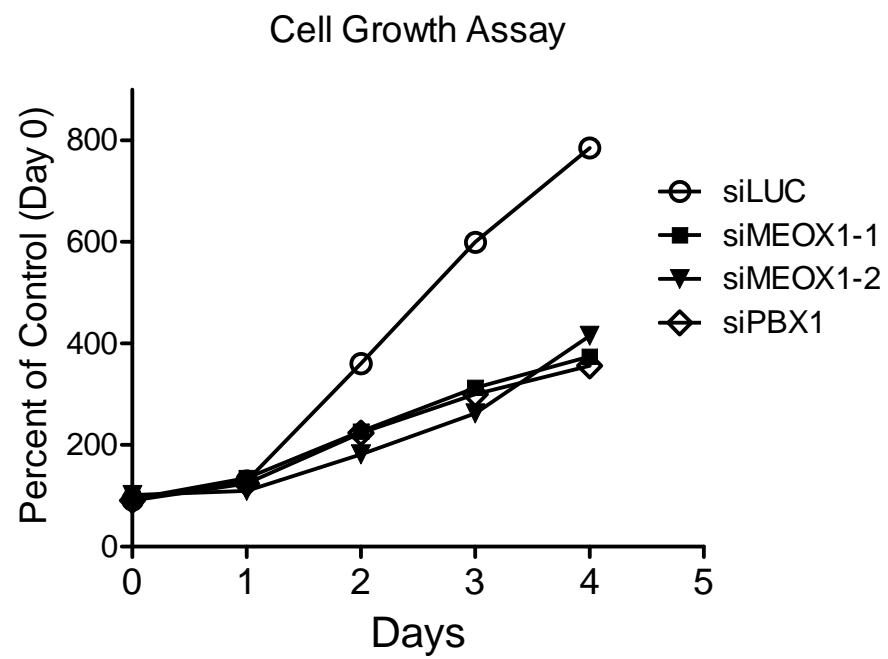

Supplement: Figure S5 — Knockdown of MEOX1 inhibits cell growth of OVCAR3 cells. A. Western blot demonstrates the knockdown efficiency of two MEOX1 siRNAs. siRNA against luciferase (siLuc) was used as a control. The cell lysate from each siRNA treatment was loaded onto two lanes of an SDS polyacrylamide gel. B. Relative cell numbers were determined by the fluorescence intensity measured at different time points compared to that measured on day 0. (PDF) [file pone.0036054.s005.pdf]

Fig. S6

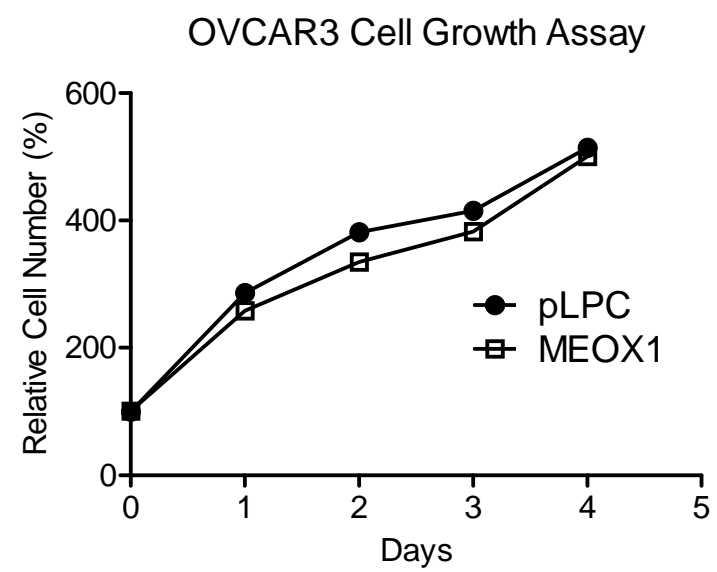

Supplement: Figure S6 — Cellular proliferation assay performed in OVCAR3 cells stably expressing MEOX1. Relative cell number was determined by the fluorescence intensity measured at different time points compared to the fluorescence intensity measured on day 0. (PDF) [file pone.0036054.s006.pdf]
